# Selective alterations of endocannabinoid system genes expression in obsessive compulsive disorder

**DOI:** 10.1038/s41398-024-02829-8

**Published:** 2024-02-26

**Authors:** Fabio Bellia, Antonio Girella, Eugenia Annunzi, Beatrice Benatti, Matteo Vismara, Alberto Priori, Fabiana Festucci, Federico Fanti, Dario Compagnone, Walter Adriani, Bernardo Dell’Osso, Claudio D’Addario

**Affiliations:** 1 Department of Bioscience and Technology for Food, Agriculture and Environment, University of Teramo, 64100 Teramo, Italy; 2 Center for Advanced Studies and Technology (CAST), University “G. D’Annunzio” of Chieti-Pescara, 66100 Chieti, Italy; 3 Department of Neuroscience, Imaging and Clinical Sciences, University “G. d’ Annunzio” of Chieti-Pescara, 66100 Chieti, Italy; 4 Department of Psychiatry, Department of Biomedical and Clinical Sciences “Luigi Sacco”, University of Milan, ASST Fatebenefratelli-Sacco, 20019 Milan, Italy; 5 “Aldo Ravelli” Center for Nanotechnology and Neurostimulation, University of Milan, Milan, Italy; 6 Department of Biotechnological and Applied Clinical Sciences, University of L’Aquila, L’Aquila, Italy; 7 Center for Behavioural Sciences and Mental Health, Istituto Superiore di Sanità, Viale Regina Elena, 299, I-00161 Rome, Italy; 8 Department of Clinical Neuroscience, Karolinska Institute, 10316 Stockholm, Sweden

**Keywords:** Neuroscience, Biomarkers

## Abstract

Obsessive Compulsive Disorder (OCD) is listed as one of the top 10 most disabling neuropsychiatric conditions in the world. The neurobiology of OCD has not been completely understood and efforts are needed in order to develop new treatments. Beside the classical neurotransmitter systems and signalling pathways implicated in OCD, the possible involvement of the endocannabinoid system (ECS) has emerged in pathophysiology of OCD. We report here selective downregulation of the genes coding for enzymes allowing the synthesis of the endocannabinoids. We found reduced DAGLα and NAPE-PLD in blood samples of individuals with OCD (when compared to healthy controls) as well as in the amygdala complex and prefrontal cortex of dopamine transporter (DAT) heterozygous rats, manifesting compulsive behaviours. Also mRNA levels of the genes coding for cannabinoid receptors type 1 and type 2 resulted downregulated, respectively in the rat amygdala and in human blood. Moreover, NAPE-PLD changes in gene expression resulted to be associated with an increase in DNA methylation at gene promoter, and the modulation of this gene in OCD appears to be correlated to the progression of the disease. Finally, the alterations observed in ECS genes expression appears to be correlated with the modulation in oxytocin receptor gene expression, consistently with what recently reported. Overall, we confirm here a role for ECS in OCD at both preclinical and clinical level. Many potential biomarkers are suggested among its components, in particular NAPE-PLD, that might be of help for a prompt and clear diagnosis.

## Introduction

Obsessive Compulsive Disorder (OCD) is a disabling condition characterised by the presence of recurrent and intrusive thoughts, images (obsessions) and urges or repetitive behaviours (compulsions) [1]. Compulsions, strongly repeated even in relatively small intervals of time and driven by obsessions, are performed in response to anxiety/distress or according to rules that must be rigidly applied. These symptoms are significantly time-consuming, distressing, and strongly impact patients’ quality of private and professional life. The worldwide prevalence of OCD is estimated to be around 2–3% [2], with the onset usually ranging within the ’30 s [1, 3, 4]. Although there is no difference in gender distribution, early onset is more common in males [5].

The neurobiology of OCD has not yet been completely defined. However, several lines of evidence support the relevance of altered serotonergic neurotransmission and selective serotonin reuptake inhibitors (SSRIs) are the first line and most effective pharmacological treatment for OCD [6]. GABAergic [7, 8], glutamatergic [9], and dopaminergic [10, 11] systems have also been considered [12]. More recently, a possible involvement of the endocannabinoid system (ECS) in the pathophysiology of OCD has emerged, primarily supported by the high presence of type 1 endocannabinoid receptor (CB1) in the prefrontal cortex (PFC), amygdala (AMY), as well as other brain areas involved in the pathology of OCD [13]. Moreover, preclinical models demonstrated how the activation of the endocannabinoid signalling directly impact OCD-related behaviours [14–16], and improve symptoms of anxiety and compulsive behaviours in patients with OCD [17–19]. Additionally, in previous papers from our group, we reported alterations in the transcription of Brain Derived Neurotrophic Factor (BDNF) [20] and Oxytocin receptor (OXTR) [21] in individuals with OCD. Of relevance, a functional cross-talk between BDNF and ECS signalling has been revealed by many reports [22–25], as well as the interactions between ECS and oxytocinergic system, of relevance in social bonding and social reward [26].

In the present paper, we aimed to explore in peripheral blood mononuclear cells (PBMCs) of subjects diagnosed with OCD the transcriptional regulation of ECS genes, namely: genes encoding for the endocannabinoid receptors (CNR1 and CNR2), synthesising enzymes N-acylphosphatidylethanolamine (NAPE)-phospholipase D hydrolase (NAPE-PLD) and diacylglycerol lipase (DAGL*α*) and degradative enzymes (fatty acid amide hydrolase (FAAH) and monoacylglycerol lipase (MAGL)). In terms of preclinical modelling, we also investigated brain regions of dopamine transporter (DAT) mutant rats, manifesting compulsive behaviour [27–29]. In the present paper we focused our attention on a specific subtype of DAT heterozygous rats. Namely, we did not use generic and mixed-asset HETs like those usually obtained by a classical HET x HET breeding. Rather, we used maternal [MAT]-HET with a DAT -/- allele of paternal origin (obtained by crossing a DAT-KO male rat with a WT female). Note that in such breeding the sperm carrying the DAT -/- allele also matures within a -/- epididymis, a condition recently suggested to entail epigenetic sequels. These are more vulnerable to the development of compulsivity compared to both wild type and generic heterozygotes, including MIX-HET rats (obtained at second filial generation by crossing MAT-HET females with KO male rats) [27]. We chose to analyse the Prefrontal Cortex (PFC) and the amygdala complex (AMY), brain regions showing a high distribution of the ECS [30], with strong connections between each other [31] and which interactions are relevant in emotions, motivations and cognitive processes [32–35]. Moreover, it has been reported that AMY inputs to the PFC and vice versa control symptoms of OCD-like behaviours [36], and this signalling is under the influence of the ECS [37].

We also investigated the possible involvement of epigenetic mechanisms, already suggested as potential molecular mechanisms in the pathogenesis of OCD [12] and which role in key genes regulation has been already reported by our and other research groups [38–40]. Of relevance in the frame of current work, higher levels of oxytocin receptor (OXTR) gene DNA methylation have been reported in individuals with OCD when compared to healthy controls [21] and therefore, we hypothesise that possible regulation of ECS genes might be connected to those of OXTR.

## Materials and methods

### Human samples

Thirty-five patients with OCD were recruited at the OCD tertiary outpatient clinic at “Luigi Sacco” University Hospital in Milan, Italy. Diagnoses were assessed by trained psychiatrists using a semi-structured interview based on DSM-5 criteria (SCID 5 research version, RV) [41]. In the case of psychiatric comorbidity, OCD had to be the primary disorder and illness severity was measured through the Yale-Brown Obsessive Compulsive Scale (Y-BOCS) [42]. Exclusion criteria were the presence of medical conditions and/or drug abuse. According to international guidelines in the field, all patients were on stable pharmacological treatment for at least one month [43]. Control subjects (CTRL, *n* = 32), matching for age (31.8 ± 7.4) and sex (Males: 60.7%; Females: 39.3%), were volunteers without any psychiatric disorder, as determined by the non-patient edition of the SCID and no positive family history for major psychiatric disorders in the first-degree relatives [44]. All subjects had given their written informed consent to participate in the study, which included using personal and clinical data and blood drawing for genotyping and methylation analysis. The local Ethics Committee “Milano Area 1” had previously approved the study protocol (protocol number 0045196/2022, dated 02/11/2022). Demographic and clinical characteristics of the study sample as well as psychotropics used by individuals with OCD are shown in Supplementary Table 1.

### Animal model

The generation of Wistar-Han DAT knock-out rats was previously described elsewhere [28] and kept in a HET breeding fashion at Istituto Italiano di Tecnologia (IIT; Genoa, Italy). Some progenitors, male DAT-KO rats were shipped to Istituto Superiore di Sanità (ISS; Rome, Italy) and bred with Wistar-Han WT females (Charles River, Italy), obtaining a new G0 of founder HET subjects [29]. Rats were maintained in a conditioned room (T 21 + /−1 °C, Relative Humidity 60 + /−10%) and were housed in pairs in Makrolon III cages with standard chow and tap water. Breeding pairs of WTxWT and KOxWT rats were realised at the same time. At delivery, on PND1, pups were culled to 5 males and 3 females. Lactating dams were WT in both groups. At weaning, on PND24, subjects were housed in pairs of non-sibling rats of the same genotype.

In this work, we analysed PFC and AMY collected from male rats sacrificed at the age of four months: *N* = 10 control rats (WT) and *N* = 10 MAT-HET rats (MAT-HET: offspring from WT mother and DAT-KO father) showing compulsive behaviours as previously reported [27]. All experimental procedures have been approved by the ISS animal welfare survey board, on behalf of the Italian Ministry of Health (formal license 937/2018-PR and 1008/2020-PR, delivered to W. A.).

The chosen *n* = 10 is larger than what usually adopted in ex vivo molecular studies. We are thus confident that no false negative errors occurred. The rats of the two groups were sacrificed on an alternating and counterbalanced order, to avoid the potential bias due to progressive disappearance of rats from the facility room. At dissection of brain areas, animal samples were given a code and molecular analyses were run in full blind concerning the rat genotype.

### Molecular analysis

#### Gene expression and DNA methylation analysis

Rats were sacrificed by decapitation and brains quickly dissected on ice. Fresh brain areas were immediately frozen in isopentane and stored at −80 °C until assays. The preparation of nucleic acids from PBMCs and rat brain regions for gene expression and DNA methylation analysis is described elsewhere [20, 45]. Primer sequences used for gene expression are reported in Supplementary Table 2. Primer sequences used for DNA methylation analysis and detailed CpG sites’ locations are reported in Supplementary Tables 3, 4, and 5.

#### Endocannabinoid levels quantification

PFC and AMY endocannabinoid anandamide [AEA] and 2-Arachidonoylglycerol [2-AG] levels were analysed by UPLC-MS/MS equipment; a mass spectrometer Qtrap 4500 (Sciex, Toronto, Ontario, Canada) coupled with Shimadzu Nexera LC20 AD system (Shimadzu, Tokyo, Japan) was used in according to previous work [46]. Briefly, samples were homogenised with deuterated internal standard (IS) by Precellys (Bertin, Montigny-le-Bretonneux, France), centrifuged and supernatant was collected and pass through OMIX C18 200 µL micro tips. Eluted samples were sent to UHPLC MS/MS analysis. Analytes were separated by BEH C18 1.8 µm 100 × 2.1 mm (Waters, Milford, Massachusetts, United States). Quantification of target analytes were performed by MultiQuant 3.0.3 software by Sciex.

### Statistical analysis

All data are expressed as the mean ± SEM, and the statistical analysis was performed with GraphPad Prism 9 software. Unpaired Mann–Whitney t-test was used to analyse gene expression and brain endocannabinoid levels. Multiple *t* test, corrected with the Sidak-Bonferroni method, was used to compare DNA methylation levels in the individual CpG sites between the groups. Spearman’s correlation analysis was used to measure the strength and direction of the relationship between the data. *p* < 0.05 was predetermined as the threshold for statistical significance.

## Results

### Human study

#### Gene expression

**Fig. 1:**
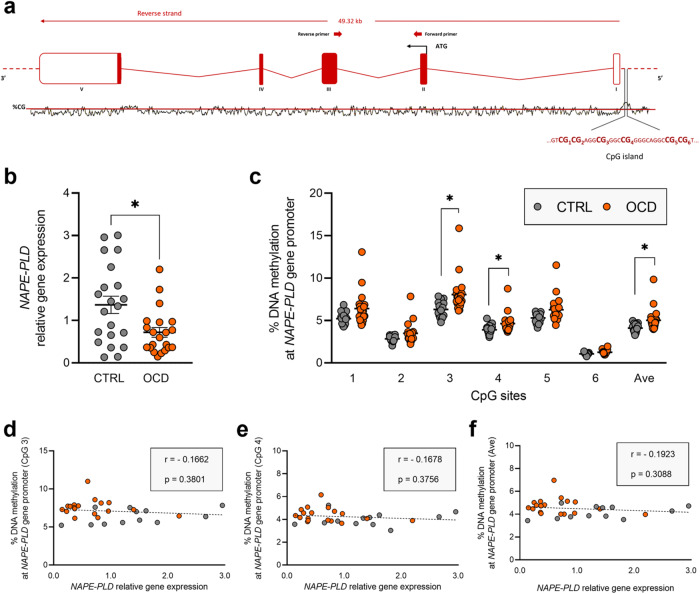
*NAPE-PLD* expression and regulation in human PBMCs. **a** Schematic representation of human *NAPE-PLD* gene. ATG is the translation start site: filled boxes represent the exons’ translated sequence. In the lower part is shown the CpG island with their sequences and the position of the CpG sites under study. In the upper part are shown the location of primers used for mRNA quantification. **b** *NAPE-PLD* relative gene expression in human PBMCs from patients diagnosed with OCD and healthy subjects (CTRL). Scattered plots represent individuals’ mRNA abundance calculated by the Delta-Delta Ct (ΔΔCt) method. *p < 0.05 Mann–Whitney test. **c** % of DNA methylation represented as scattered dot plots for the individual CpG sites under study as well as for the average (Ave) of the 6 CpG sites. Significant differences are indicated (Bonferroni corrected), *p < 0.05. Values are reported in Supplementary Table 7. Correlation analysis between *NAPE-PLD* relative gene expression and % of DNA methylation. The % of DNA methylation refers to the CpG 3 (**d**), CpG 4 (**e**), and the average (Ave) (**f**) of the 6 CpG sites under study, while the relative gene expression refers to individuals’ 2^^-ΔΔCt^ values, Y and X bars respectively. Data were compared by Spearman’s rank correlation coefficient: Spearman’s r and *p* value are reported inside the boxes.

**Fig. 2:**
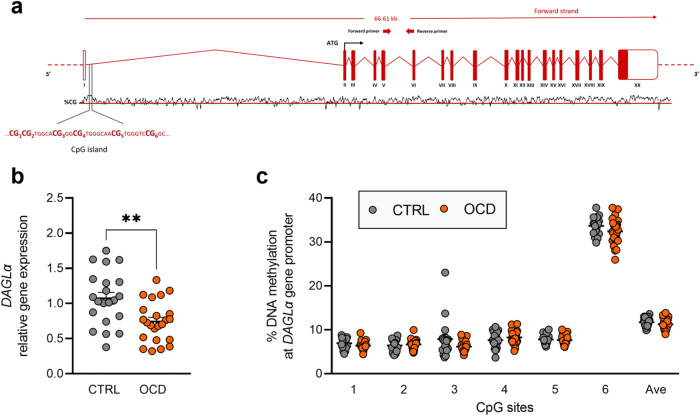
*DAGLα* expression and regulation in human PBMCs. **a** Schematic representation of human *DAGLα* gene. ATG is the translation start site: filled boxes represent the exons’ translated sequence. In the lower part is shown the CpG island with their sequences and the position of the CpG sites under study. In the upper part are shown the location of primers used for mRNA quantification. **b** *DAGLα* relative gene expression in human PBMCs from patients diagnosed with OCD and healthy subjects (CTRL). Scattered plots represent individuals’ mRNA abundance calculated by the Delta-Delta Ct (ΔΔCt) method. **p < 0.01 Mann–Whitney test. **c** % of DNA methylation represented as scattered dot plots for the individual CpG sites under study as well as for the average (Ave) of the 6 CpG sites under study. Values are reported in Supplementary Table 8.

**Fig. 3:**
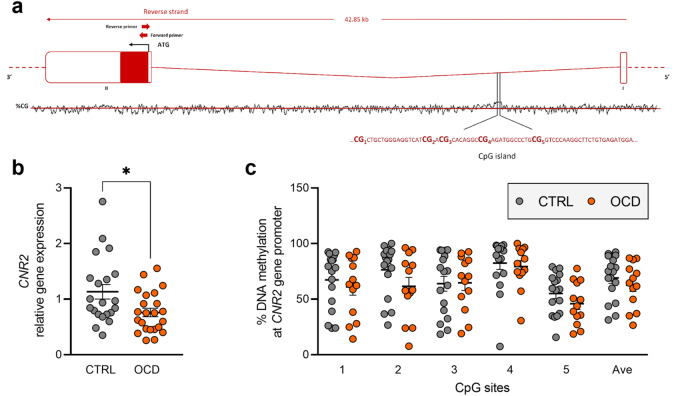
*CNR2* expression and regulation in human PBMCs. **a** Schematic representation of human *CNR2* gene. ATG is the translation start site: filled boxes represent the exons’ translated sequence. In the lower part is shown the CpG island with their sequences and the position of the CpG sites under study. In the upper part are shown the location of primers used for mRNA quantification. **b** *CNR2* relative gene expression in human PBMCs from patients diagnosed with OCD and healthy subjects (CTRL). Scattered plots represent individuals’ mRNA abundance calculated by the Delta-Delta Ct (ΔΔCt) method. *p < 0.05 Mann–Whitney test. **c** % of DNA methylation represented as scattered dot plots for the individual CpG sites under study as well as for the average (Ave) of the 5 CpG sites under study. Values are reported in Supplementary Table 9.

The first result of this study is a different expression between individuals with OCD and healthy controls in genes encoding specific endocannabinoids’ synthesise hormones and receptors. Specifically, we observed a significant reduced expression of *NAPE-PLD* (CTRL: 1.369 ± 0.203; OCD: 0.721 ± 0.115, *p* = 0.0253) (Fig. 1b), *DAGLα* (CTRL: 1.074 ± 0.084; OCD: 0.742 ± 0.060, *p* = 0.0056) (Fig. 2b), and *CNR2* (CTRL: 1.091 ± 0.118; OCD: 0.758 ± 0.069, *p* = 0.0263) (Fig. 3b) in individuals with OCD compared to healthy individuals. There was no difference between the two groups for *CNR1, FAAH*, and *MAGL* (Supplementary Table 6). Interestingly, when stratifying the data considering subjects’ characteristics, we observed *DAGLα* downregulation in females (CTRL: 1.064 ± 0.130; OCD: 0.600 ± 0.079, *p* = 0.009), but not in males (Supplementary Fig. 2a, b). No differences were observed in other ECS genes under study (Supplementary Figs. 1 and 3).

#### DNA methylation

We analysed DNA methylation levels at the promoter region of the genes emerged to be differentially modulated in OCD compared to healthy individuals. We studied 6 CpG sites at the *NAPE-PLD* promoter region (Fig. 1a), observing significant differences between the groups. In particular, individuals with OCD show significant increased levels of DNA methylation at CpG 3 (CTRL: 6.311 ± 0.223; OCD: 8.059 ± 0.476, *p* = 0.0016), CpG 4 (CTRL: 3.915 ± 0.132; OCD: 4.646 ± 0.254, *p* = 0.0486), and at the average (AVE) of the 6 CpG sites considered for the analysis (CTRL: 4.112 ± 0.121; OCD: 5.025 ± 0.301, *p* = 0.0201) (corrected p values using the Sidak-Bonferroni method for the multiple comparisons) (Fig. 1c and Supplementary Table 7). At the *DAGLα* promoter region (Fig. 2a), in all the 6 CpG sites considered for the analysis, we did not observe any difference between the two study sample groups (Fig. 2b and Supplementary Table 8). At the *CNR2* promoter region (Fig. 3a), in all the 5 CpG sites under study, we did not observe difference between the two groups (Fig. 3b and Supplementary Table 9).

When stratifying the data considering subjects’ characteristics, we observed increased DNA methylation levels at the *NAPE-PLD* CpG sites 3, 4, 5, and in the Average selectively in male patients (Supplementary Fig. 1f). No differences were observed for *DAGLα* and *CNR2* (Supplementary Figs. 2 and 3).

#### Correlation analysis

Correlating the % of DNA methylation levels at the CpG sites observed to be differentially modulated between the two groups under study with the relative gene expression (2^-ΔΔCt^ values), we observed a tendency to anti-correlation for *NAPE-PLD*, both for the individuals with OCD and the healthy individuals, but without reaching statistical significance (Fig. 1d–f). The correlation analysis between DNA methylation and gene expression performed for *DAGLα* and *CNR2* did not reveal any relationship between mRNA abundance and DNA methylation levels (AVE) at the gene promoter regions (data not shown).

We also correlated the gene expression and the average % of DNA methylation levels of the genes under study with the clinical characteristics of the patients, considering the duration of illness (expressed in years) and the YBOCS score. No correlation was observed for *DAGLα* and *CNR2*, while an anticorrelation was observed between *NAPE-PLD* gene expression and the years of disease (Spearman’s *r* = −0.5695, *p* = 0.0232) (Supplementary Fig. 1d).

Correlating the relative expression of the different studied genes, we observed that all the subjects display a robust direct correlation between *DAGLα* and *NAPE-PLD* (Spearman’s *r* = 0.553, *p* < 0.001) (Supplementary Fig. 4a). The correlation remains statistically significant when we look at those between *DAGLα* and *NAPE-PLD* with *CNR2* (*p* < 0.001 for both), between *CNR2* and *FAAH* (*p* = 0.0163), *DAGLα* and *NAPE-PLD* (*p* < 0.001), and *NAPE-PLD* and *MAGL* (*p* = 0.021), but lose significance, as expected, when we consider correlations between *DAGLα* and *MAGL*, and also between *NAPE-PLD* and *FAAH* (Supplementary Fig. 4a). When we considered separating individuals with OCD from healthy subjects, we observed that only the correlations between *MAGL* (Spearman’s *r* = 0.539, *p* = 0.012) and *DAGLα* (Spearman’s *r* = 0.456, *p* = 0.043) with *NAPE-PLD* remained statistically significant (Supplementary Fig. 4b).

**Fig. 4:**
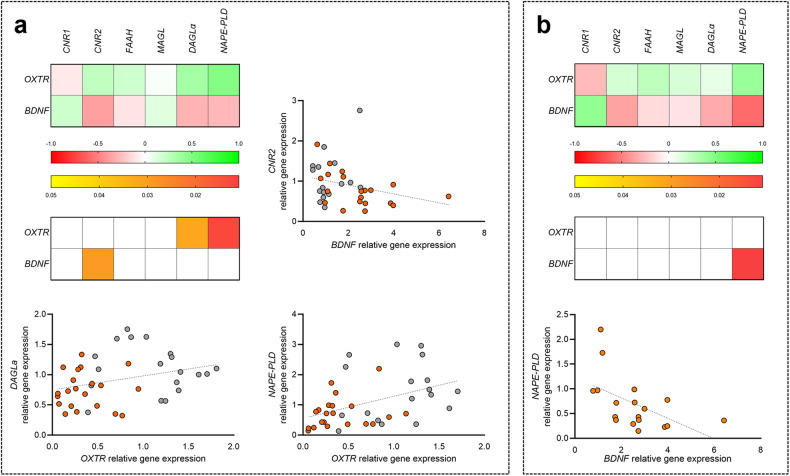
Correlation analysis between ECS components and *OXTR* and *BDNF* in human PBMCs. Heat maps representing the correlation analysis between ECS components and previously studied *OXTR* and *BDNF* genes expression, in the overall human population under study (**a**) or in OCD patients alone (**b**). Cells filled in green to red gradient of the heat maps (upper part) represent Spearman’s r; cells filled in yellow to red gradient (lower part) represent *p* values (empty cells represent *p* values greater than 0.05). X/Y graphs represent the statistically significant individual correlations.

We then correlated the expression of studied ECS genes with *BDNF* and *OXTR* genes, previously investigated in the same subjects (see the papers [20, 21] for detailed investigation). Considering the overall population, we noted a correlation of *DAGLα* (Spearman’s *r* = 0.351, *p* = 0.027) and *NAPE-PLD* (Spearman’s *r* = 0.482, *p* = 0.002) with *OXTR*, together with an anticorrelation between *CNR2* and *BDNF* expression (Spearman’s *r* = −0.376, *p* = 0.024) (Fig. 4a). Considering the individuals with OCD alone, we observed an anticorrelation between *NAPE-PLD* and *BDNF* gene expression (Spearman’s *r* = −0.583, *p* = 0.011) (Fig. 4b).

#### Animal model

##### Gene expression

**Fig. 5:**
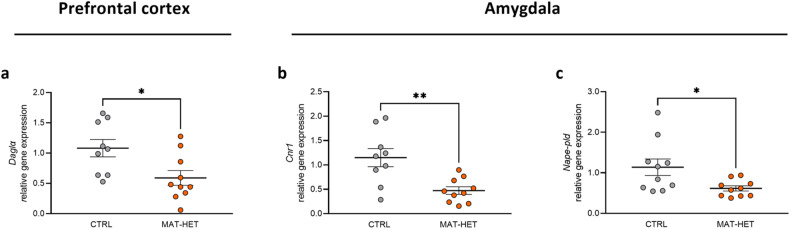
*Daglα*, *Cnr1* and *Nape-pld* expression in selective rat brain regions. *Daglα* (**a**), *Cnr1* (**b**), and *Nape-pld* (**c**) relative gene expression in rat PFC and AMY. Scattered plots represent individuals’ mRNA abundance calculated by the Delta-Delta Ct (ΔΔCt) method. *p < 0.05, **p < 0.01 Mann–Whitney test.

We analysed the expression of genes encoding for the ECS in the PFC and AMY of MAT-HET and CTRL rats. At the PFC, MAT-HET rats show a decrease in *Daglα* mRNA abundance compared to controls (CTRL: 1.081 ± 0.144; MAT-HET: 0.589 ± 0.121, *p* = 0.0161, Mann–Whitney test) (Fig. 5a). In the same brain region, we did not observe any difference between the groups for *Nape-pld* mRNA abundance (CTRL: 1.025 ± 0.073; MAT-HET: 1.140 ± 0.261, *p* = 0.968), *Cnr1* (CTRL: 1.142 ± 0.176; MAT-HET: 1.011 ± 0.088, *p* = 0.684), *Cnr2* (CTRL: 1.214 ± 0.253; MAT-HET: 1.067 ± 0.222, *p* = 0.720), *Faah* (CTRL: 1.107 ± 0.140; MAT-HET: 1.196 ± 0.336, *p* = 0.481), and *Magl* (CTRL: 1.088 ± 0.156; MAT-HET: 1.195 ± 0.310, *p* = 0.720) (Supplementary Table 10). In the AMY, we did not observe a difference in *Daglα* gene expression (CTRL: 1.070 ± 0.168; MAT-HET: 1.360 ± 0.200, *p* = 0.079) (Supplementary Table 10), while *Nape-pld* expression was reduced in MAT-HET (0.616 ± 0.064) respect to the control group (1.138 ± 0.203, *p* = 0.028) (Fig. 5c). Together with *Nape-pld*, a downregulation of C*nr1* was observed in MAT-HET compared to the CTRL group (CTRL: 1.148 ± 0.186; MAT-HET: 0.472 ± 0.078, *p* = 0.004) (Fig. 5b). No difference between groups was observed for *Cnr2* (CTRL: 1.190 ± 0.249; MAT-HET: 1.332 ± 0.283, *p* = 0.842), *Faah* (CTRL: 1.196 ± 0.248; MAT-HET: 0.872 ± 0.135, *p* = 0.497), and *Magl* (CTRL: 1.192 ± 0.265; MAT-HET: 1.361 ± 0.265, *p* = 0.661) (Supplementary Table 10).

##### DNA methylation

We analysed DNA methylation levels in the promoter region of *Nape-pld, Daglα*, and *Cnr1* genes, selectively in the brain region in which MAT-HET rats showed altered gene expression respect the wild-type rats. For all the three genes, we did not observe difference in DNA methylation levels in all the CpG sites under study between the two groups (Supplementary Fig. 5).

##### Endocannabinoids levels

**Fig. 6:**
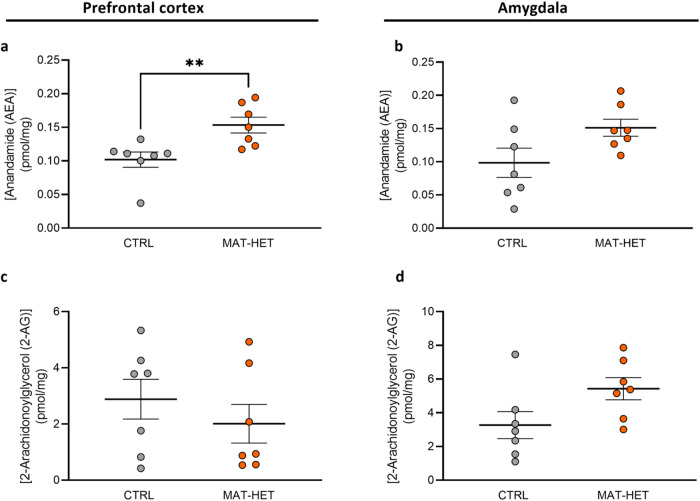
Endocannabinoids content in selective rat brain regions. Anandamide (AEA) (**a**, **b**) and 2-Arachinonoylglycerol (2-AG) (**c**, **d**) levels in MAT-HET and CTRL rats’ PFC and AMY. Scattered plots represent individuals’ endocannabinoids levels expressed as pmol/mg. **p < 0.01 Mann–Whitney test.

Looking at the endocannabinoid levels, we observed in the PFC an increase in AEA levels in MAT-HET (0.153 ± 0.012) with respect to the control group (0.102 ± 0.011) (*p* = 0.0.002) (Fig. 6a). In the same region, there is no difference between the two groups concerning 2-AG levels (CTRL: 2.884 ± 0.708; MAT-HET: 2.012 ± 0.0.688; p = 0.620) (Fig. 6b). In the AMY, we observed a little increase in AEA levels in MAT-HET (0.151 ± 0.013) with respect to the control group (0.098 ± 0.022) but without reaching statistical significance (*p* = 0.128) (Fig. 6c) and the same tendency for 2-AG, with MAT-HET displaying higher levels (CTRL: 3.269 ± 0.803; MAT-HET: 5.429 ± 0.655, *p* = 0.053) (Fig. 6d).

##### Correlation analysis between molecular data

We correlated AEA and 2-AG levels with synthesis’ (*Nape-pld* and *Daglα*) enzymes gene expression in the two brain regions. In the PFC, we did not observe a correlation either for *Nape-pld* with AEA levels (Spearman’s *r* = −0.1297, *p* = 0.660) or between *Daglα* and 2-AG levels (Spearman’s *r* = −0.0055, *p* > 0.99) (Supplementary Table 11). In the AMY, we observed an anti-correlation-like between *Nape-pld* and AEA levels (Spearman’s *r* = −0.4422, *p* = 0.114). No relationship for *Daglα* with 2-AG levels was observed (Spearman’s *r* = −0.1473, *p* = 0.616) (Supplementary Table 11).

Moreover, we observed a strong direct correlation for all the genes investigated except for *Daglα*, which does not reach statistical significance with any of the other genes in the PFC (Supplementary Fig. 6a). All these correlations are also observed in the AMY (Supplementary Fig. 6b), also if less strong than those observed in the PFC, and with *Daglα* showing a weak anti-correlation with *Cnr1*. As for the human samples, we also tried to correlate ECS genes expression with previously analysed (Supplementary Table 12) *Bdnf* and *Oxtr* gene expression, observing a direct significant correlation only between *Oxtr* and *Magl* expression in the PFC of MAT-HET and CTRL rats (Supplementary Fig. 7).

## Discussion

In the present study we analysed the gene regulation of the ECS using genomic DNA and total RNA extracted from PBMCs of individuals with OCD and healthy subjects, as well as from selective brain regions of rats predisposed to develop compulsivity-like behaviour. The first relevant result we observed is the downregulation in individuals with OCD when compared to healthy controls of both genes encoding DAGLα and NAPE-PLD, the enzymes synthesising respectively the endocannabinoids 2-AG and AEA [47, 48], as well as of *CNR2*, the gene encoding for the cannabinoid receptor type 2. As already suggested, PBMCs might mirror the ECS status of the central nervous system (CNS) [49] and investigations from our and other research groups have already reported selective modulation of ECS genes regulation analysing PBMCs in different psychiatric and neurological disorders [50, 51]. Consistent with the results of the present paper the reduction of NAPE-PLD, DAGL, and CB2 protein levels in PBMCs of patients with a first episode of psychosis compared to healthy controls has been previously reported [52]. Obsessive thoughts are actually products of the person’s mind, thus kind of psychosis and it should be noted that there is a relationship between OCD and psychosis symptoms [53] which are more common in individuals with OCD when compared with the rest of the population [54, 55]. *CNR2* expression resulted also significantly reduced in PBMCs of individuals with schizophrenia [56, 57] even if others reported an increase in mRNA levels of this receptor in schizophrenia [58] as well as in autistic children [59]. It should be recalled that activation of *CNR2*, primarily expressed in immune cells, such as PBMCs [30], decrease inflammation in many disorders, including those associated with neuroinflammation [60]. It is known that CB1 receptors mainly act in the CNS, whereas CB2 receptors do it mainly at the peripheral level, and even if *CNR1* is expressed in human PBMCs, its levels are much lower than *CNR2* [61] and this might be why we have not observed a modulation of this gene expression in our study. OCD can sometimes have similar symptoms of Autism Spectrum Disorder and frequently these conditions occur together [62]. The role of ECS in ASD [63] and the transcriptional regulation of ECS components in ASD has been investigated and, consistently with our observations in OCD, a reduction in *NAPE-PLD* mRNA levels has been reported in PBMCs of autistic children [59]. However, in the same study the authors also reported an increase in *CNR2* gene expression in these children. Moreover, a reduced eCB signalling was also observed in autistic children and in the ASD animal model [64]. As already mentioned in the introduction section, we previously observed alterations in *BDNF* [20] and *OXTR* [21] mRNA levels, when compared to healthy controls, in the same population of individuals with OCD analysed in this paper. We here reported a direct significant correlation between *OXTR* and both *DAGLα* and *NAPE-PLD* mRNA levels in the population under study. Reduced mRNA levels of the enzymes for eCBs synthesis are correlated with reduced levels of *OXTR* partially in agreement with what reported about the inhibition of eCB signalling connected with the blockade of OXTR in reducing social reward [26] which is altered also in individuals with OCD [65]. We also observed a significant inverse correlation between *BDNF* and *CNR2* gene expression, which in turn might suggest their role as potential biomarkers of the disorder.

Based on the results on ECS genes expression, we investigated the possible role of epigenetic mechanisms in the modulation of those genes that emerged to be altered in individuals with OCD. In detail, we focused our attention on the study of DNA methylation at selective CpG sites present in the CpG islands at *NAPE-PLD*, *DAGLα* and *CNR2* gene promoters. *NAPE-PLD* was the only gene promoter where we observed a significant difference in DNA methylation levels between individuals with OCD and healthy controls. In particular, a higher DNA methylation levels at the CpG sites named 3 and 4, as well as in the average of the 6 CpG sites under study emerged. Assuming that an increase in DNA methylation involves a less-accessible DNA to the transcription machinery [66], this is in agreement with the reduced gene expression even if there is no significant correlation between mRNA levels and DNA methylation. To the best of our knowledge, this is the first study monitoring the role of the epigenetic mark at the level of *NAPE-PLD* gene in human samples. After stratification of the data on genes expressions and DNA methylation, based on gender, pharmacological treatment, duration, and severity of the disease, we first observed that none of the genes transcriptional regulation was affected by the pharmacotherapy (considering monotherapy with antidepressants or in augmentation), however it should be noted that there were no drug naive subjects participating to the study. Of interest, a significant inverse correlation between *NAPE-PLD* gene expression and the progression of the disease emerged, and this data was also partially corroborated by the direct correlation between DNA methylation at gene promoter and the duration of the disease. Indeed, this evidence further supports the possible role of *NAPE-PLD* gene as a potential biomarker in OCD and especially for its epigenetic regulation that might be environmentally switched. Another interesting data come out after stratification is that the reduction in mRNA levels of DAGLα appeared to be relevant in females but not in males, whereas when considering *NAPE-PLD* this effect was more evident in males than females.

We thus evaluated the transcriptional regulation of ECS gene components in brain regions of an animal model resembling OCD [27]. Again, comparing DAT-HET rats to WT ones we reported a down regulation of *Daglα* in the PFC and of *Nape-pld* and *Cnr1* in the AMY complex, whereas no changes have been observed in both brain regions for mRNA levels of eCB degrading enzymes, likewise in the human samples. No differences were found, as well for *Cnr2* which, as mentioned above, was more expressed at the peripheral level. Thus, in our “compulsive” rats we have reduced levels of *Cnr1* expression and reduced levels of the enzymes accounting for eCBs synthesis. Unexpectedly, we found higher levels of AEA that however can be seen as a temporary compensation mechanism needed to activate the lower quantity of CB1 receptors. The analysis of *Oxtr* and *Bdnf* mRNA levels in rat brain regions did not reveal any differences between the two groups of rats, suggesting a very specific involvement of ECS. CB1 receptors are differentially expressed in the brain at low levels in some brain regions like the nucleus accumbens and the ventral tegmental area, and with moderate to very high levels in others like the PFC and the AMY [67–70]. Considering the direct connection between the PFC and the AMY and its role in controlling anxiety and fear responses, such data are suggesting a relevant role for CB1 actions in emotional processes [71]. CB1 receptor agonist WIN55,212-2 was observed to reduce compulsive behaviours in mice, reducing the number of buried marbles [14], suggesting the ECS as a target for drugs in modulating compulsive behaviour. In a randomised trial with nabilone, a CB1 agonist, Kayser and colleagues observed an improvement of YBOCS scores in individuals with OCD when the molecule was combined with cognitive behavioural therapy with exposure and response prevention (EX/RP), resulting in an improvement twice higher than EX/RP alone [18, 72].

Moreover, also the study of DNA methylation at *Daglα*, *Nape-pld* and *Cnr2* gene promoters show that this epigenetic mark is not involved in the modulation of their transcription, suggesting that other epigenetic mechanisms might be involved.

### Study limitations

First of all, human samples were collected in a single time-point. Considering the epigenetic modulation of gene expression, a longitudinal approach might be relevant to better understand the changes of the transcriptional regulation according to modification in the pharmacotherapy or the symptomatology. Another limitation might be represented that the animal model used in this study cannot be considered an animal model of OCD, however rats clearly show compulsive behavioural features [27]. We also have to consider that in the preclinical study we used only male rats. Considering the higher predisposition of female individuals to develop psychiatric and mood disorders [73], female individuals should be also considered in order to look at sex-specific differences. However, in this case also the ovarian hormone fluctuations over the oestrous cycle should be evaluated, as it was already demonstrated how the different oestrous cycle’s phases differentially exhibit anxiety-like behaviours through chromatin organisation fluctuation in the brain cells [74, 75]. Future studies should address these limitations, evaluating all the possible external factors that may contribute to the modulation of these epigenetic mechanisms.

## Conclusions

Overall, our study confirmed the involvement of the ECS in the pathogenesis of OCD at both preclinical and clinical level. So far, serotoninergic medications are the most common and effective pharmacological treatments available to attenuate OCD symptoms, although some patients do not show an adequate response. Therefore, the investigation of other systems apart from the serotoninergic one is needed and warranted. CB1 and CB2 receptors can regulate the release of neurotransmitters that have a pathogenic role in OCD, such as serotonin, dopamine, GABA, and glutamate. From this perspective, based on present data, the ECS could be a promising target for new therapy approaches. However, further studies are warranted to better understand the role of these pathways in the development and manifestation of OCD.

### Supplementary information

Supplementary legends

Supplementary materials

Supplementary Figure 1

Supplementary Figure 2

Supplementary Figure 3

Supplementary Figure 4

Supplementary Figure 5

Supplementary Figure 6

Supplementary Figure 7

## Data Availability

Data are available upon request.
