# Supplementary material for: Selective alterations of endocannabinoid system genes expression in obsessive compulsive disorder
Source: Transl Psychiatry. 2024 Feb 26;14:118. doi: 10.1038/s41398-024-02829-8 (PMC10897168; doi:10.1038/s41398-024-02829-8)
Supplement: Supplementary file 1 — Supplementary legends [file 41398_2024_2829_MOESM1_ESM.docx]

**Supplementary Figures:**

**Supplementary Figure 1** - *NAPE-PLD* relative gene expression in human PBMCs from patients diagnosed with OCD and healthy individuals (CTRL) stratified based on sex (**a**: men, **b**: women) and drug therapy (**c**). % of DNA methylation at gene promoter stratified based on sex (**f**: men, **g**: women) and drug therapy (**h**); dashed lines indicate CTRL’s DNA methylation levels for the individual CpG site. X/Y graphs represent correlation between years of disease and Y-BOCS score with gene expression (**d** and **e**) and DNA methylation (**i** and **j**). Monotherapy = antidepressants; Multitherapy = antidepressants, antipsychotics, mood stabilizers, benzodiazepines.

**Supplementary Figure 2** - *DAGLα* relative gene expression in human PBMCs from patients diagnosed with OCD and healthy individuals (CTRL) stratified based on sex (**a**: men, **b**: women) and drug therapy (**c**). % of DNA methylation at gene promoter stratified based on sex (**f**: men, **g**: women) and drug therapy (**h**); dashed lines indicate CTRL’s DNA methylation levels for the individual CpG site. X/Y graphs represent correlation between years of disease and Y-BOCS score with gene expression (**d** and **e**) and DNA methylation (**i** and **j**). Monotherapy = antidepressants; Multitherapy = antidepressants, antipsychotics, mood stabilizers, benzodiazepines.

**Supplementary Figure 3** - *CNR2* relative gene expression in human PBMCs from patients diagnosed with OCD and healthy individuals (CTRL) stratified based on sex (**a**: men, **b**: women) and drug therapy (**c**). % of DNA methylation at gene promoter stratified based on sex (**f**: men, **g**: women) and drug therapy (**h**); dashed lines indicate CTRL’s DNA methylation levels for the individual CpG site. X/Y graphs represent correlation between years of disease and Y-BOCS score with gene expression (**d** and **e**) and DNA methylation (**i** and **j**). Monotherapy = antidepressants; Multitherapy = antidepressants, antipsychotics, mood stabilizers, benzodiazepines.

**Supplementary Figure 4** - Heat maps representing the correlation analysis between ECS genes in the overall population (**a**) and in OCD patients alone (**b**). Cells filled in green to red gradient of the heat maps (upper part) represent Spearman’s r; cells filled in yellow to red gradient (lower part) s represent p values (empty cells represent p values greater than 0.05). X/Y graphs represent the individual correlation.

**Supplementary Figure 5** - % of DNA methylation at *Daglα* (**a**) gene promoter in the PFC, *Cnr1* (**b**) and *Nape-pld* (**c**) gene promoters in the AMY of MAT-HET and control (CTRL) rats. Scattered plots represent the rats’ % of DNA methylation for the individual CpG sites under study as well as for the average (Ave) of the CpG sites under study.

**Supplementary Figure 6** - Heat maps representing the correlation analysis between the individuals’ gene expression in the PFC (**a**) and AMY (**b**) of CTRL and MAT-HET rats, or MAT-HET rats alone (**c** and **d**). Cells filled in green to red gradient of the heat maps (lower part) represent Spearman’s r; cells filled in yellow to red gradient (upper part) s represent p values (empty cells represent p values greater than 0.05).

**Supplementary Figure 7** - Heat maps representing the correlation analysis between ECS components and previously studied *Oxtr* and *Bdnf* genes expression in the PFC (**a**) and AMY (**b**) of CTRL and MAT-HET rats. Cells filled in green to red gradient of the heat maps (upper part) represent Spearman’s r; cells filled in yellow to red gradient (lower part) represent p values (empty cells represent p values greater than 0.05).

**Supplementary Tables:**

**Supplementary Table 1** - Socio-demographic and clinical features of the subjects under study.

**Supplementary Table 2** - Primer sequences used for gene expression analysis with qRT-PCR in PBMCs from OCD subjects and healthy individuals (top), and in brain regions from DAT-HET and control rats (bottom).

**Supplementary Table 3** - Details of sequences and primers employed for DNA methylation analysis in human (top) and rat (bottom) samples. Highlighted in bold the individual CpG sites considered for the analysis.

**Supplementary Table 4** - Genomic coordinates of the CpG sites analysed in Human *DAGLα*, *NAPE-PLD* and *CNR2* gene promoter regions.

**Supplementary Table 5** - Genomic coordinates of the CpG sites analysed in Rat *Daglα*, *Nape-pld* and *Cnr1* gene promoter regions.

**Supplementary Table 6** - ECS components gene expression in human PBMCs from patients diagnosed with OCD and healthy subjects (CTRL). *p < 0.05, **p < 0.01 Mann-Whitney test.

**Supplementary Table 7** - Groups’ mean of DNA methylation in human PBMCs from patients diagnosed with OCD and healthy subjects (CTRL) for the individual CpG sites analysed in *NAPE-PLD* gene promoter region.

**Supplementary Table 8** - Groups’ mean of DNA methylation in human PBMCs from patients diagnosed with OCD and healthy subjects (CTRL) for the individual CpG sites analysed in *DAGLα* gene promoter region.

**Supplementary Table 9** - Groups’ mean of DNA methylation in human PBMCs from patients diagnosed with OCD and healthy subjects (CTRL) for the individual CpG sites analysed in *CNR2* gene promoter region.

**Supplementary Table 10** - ECS components gene expression in the prefrontal cortex and amygdala of MAT-HET and control (CTRL) rats. *p < 0.05, **p < 0.01 Mann-Whitney test.

**Supplementary Table 11** - Spearman’s r and p value of correlation between gene expression and endocannabinoid levels of CTRL and MAT-HET rats.

**Supplementary Table 12** - Relative gene expression of *Oxtr* and *Bdnf* in the prefrontal cortex and amygdala of CTRL and MAT-HET rats. Mann-Whitney test.
