# Supplementary material for: Selective alterations of endocannabinoid system genes expression in obsessive compulsive disorder
Source: Transl Psychiatry. 2024 Feb 26;14:118. doi: 10.1038/s41398-024-02829-8 (PMC10897168; doi:10.1038/s41398-024-02829-8)
Supplement: Supplementary file 2 — Supplementary materials [file 41398_2024_2829_MOESM2_ESM.docx]

**Supplementary Table 1:**


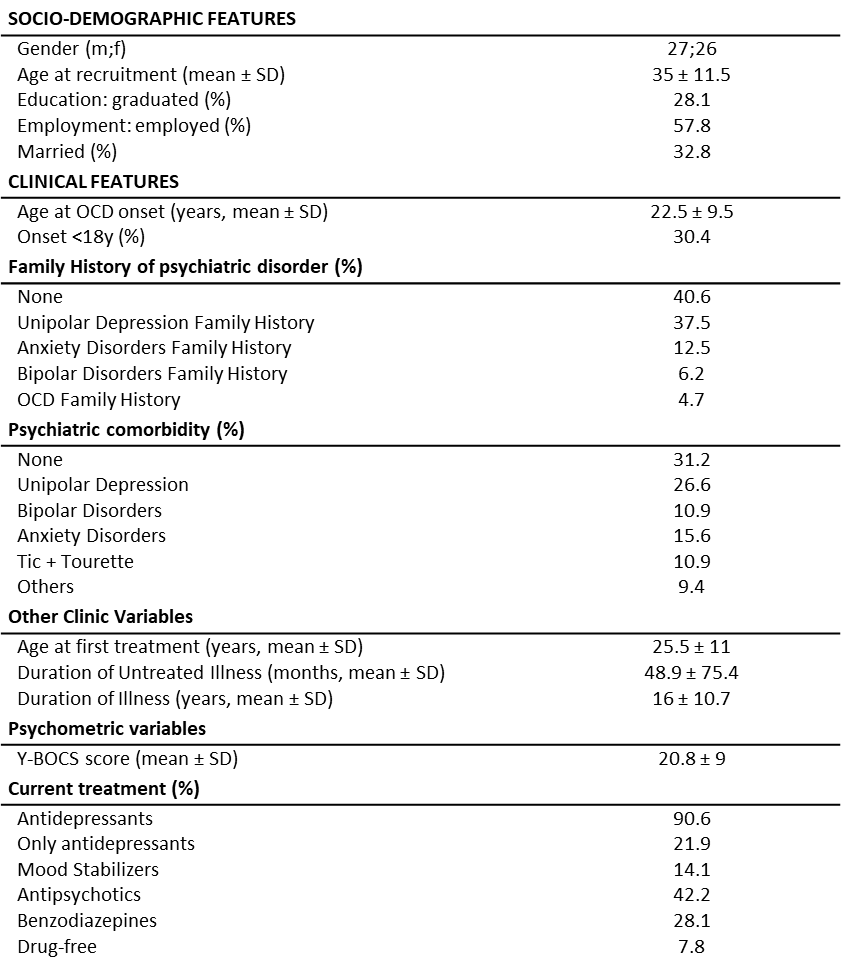


**Supplementary Table 2:**

| **Gene** | **Sequence** | |
| --- | --- | --- |
| **Human** | **Forward** | **Reverse** |
| *GAPDH* | CAGCCTCAAGATCATCAGCA | TGTGGTCATGAGTCCTTCCA |
| *β -ACT* | GACCCAGATCATCAGCA | CCATCACGATGCCAGTGG |
| *CNR1* | CCTTTTGCTGCCTAAATCCAC | CCACTGCTCAAACATCTGAC |
| *CNR2* | AAGGATGGCTTGGATTCC | ATGCAAAGACCACACTGG |
| *FAAH* | CCCAATGGCTTAAAGGACTG | ATGAACCGCAGACACAAC |
| *MAGL* | ATGCAGAAAGACTACCCTGGGC | TTATTCCGAGAGAGCACGC |
| *DAGLα* | GCCACCAAGAGGAGGCAGCG | CCGCGTGCAGCAGAGGAACA |
| *NAPE-PLD* | TTGTGAATCCGTGGCCAACATGG | TACTGCGATGGTGAAGCACG |
| **Rat** |  |  |
| *Gapdh* | AGACAGCCGCATCTTCTTGT | CTTGCCGTGGGTAGAGTCAT |
| *β-Act* | AGATCAAGATCATTGCTCCTCCT | ACGCAGCTCAGTAACAGTCC |
| *Cnr1* | TTCCACCGTAAAGACAGCCC | TCCACATCAGGCAAAAGGCC |
| *Cnr2* | TTGACCGATACCTATGTCTGTGC | TGCTTTCCAGAGGACATACCC |
| *Faah* | TGACAACTATACCATGCCCAGC | TAAGAAGGGAATCAGCGTGTGG |
| *Magl* | ACGTGAACACCGTCCAGAAG | TTGGCAGCAAGGACCTTCAA |
| *Daglα* | ATTCTCTCCTTCCTCCTGC | ATTTGGGCTTGGTGCTTCG |
| *Nape-pld* | TGTCCCGGGTTCCAAAGAGGAGC | ACCATCAGCGTCGCGTGTCC |

**Supplementary Table 3:**


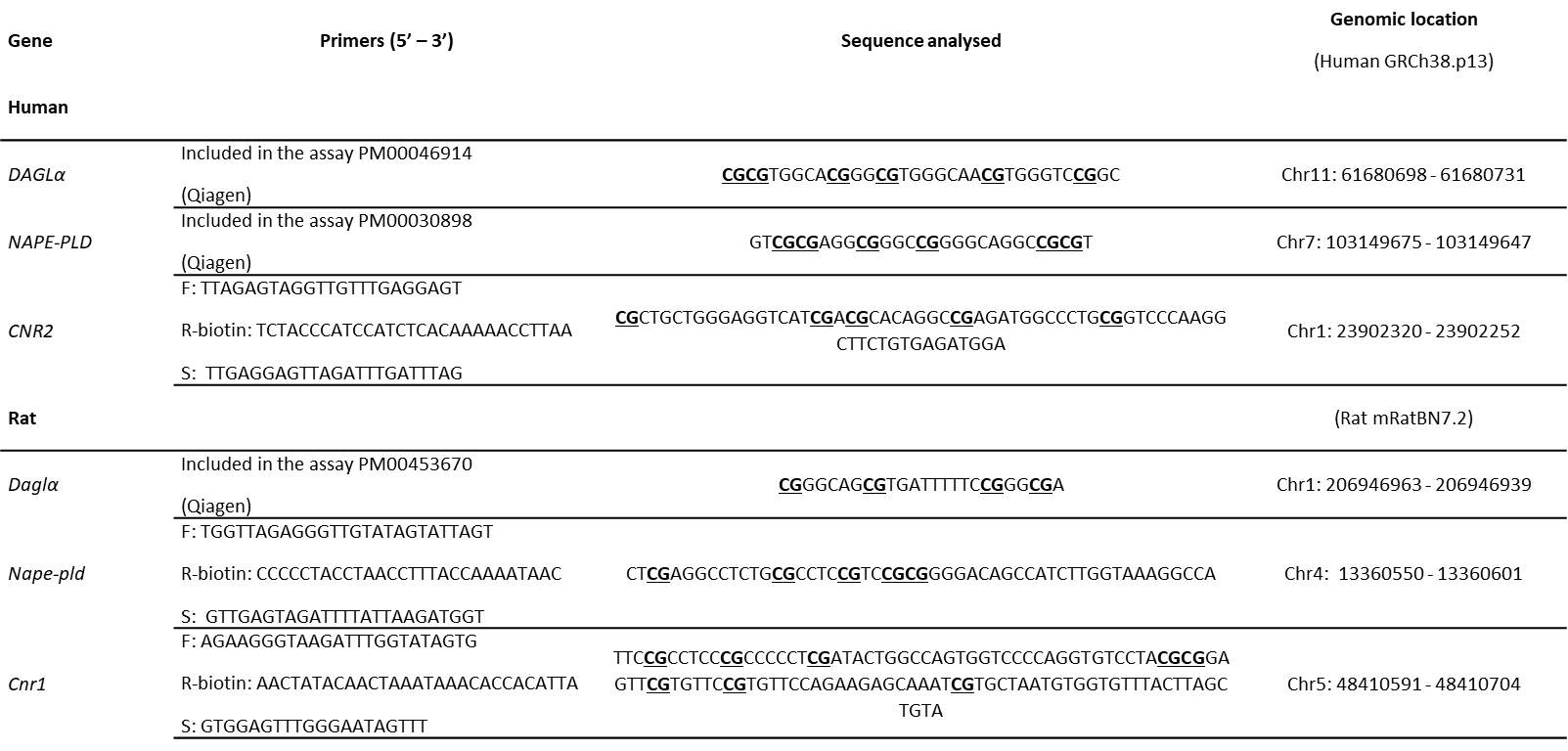


**Supplementary Table 4:**


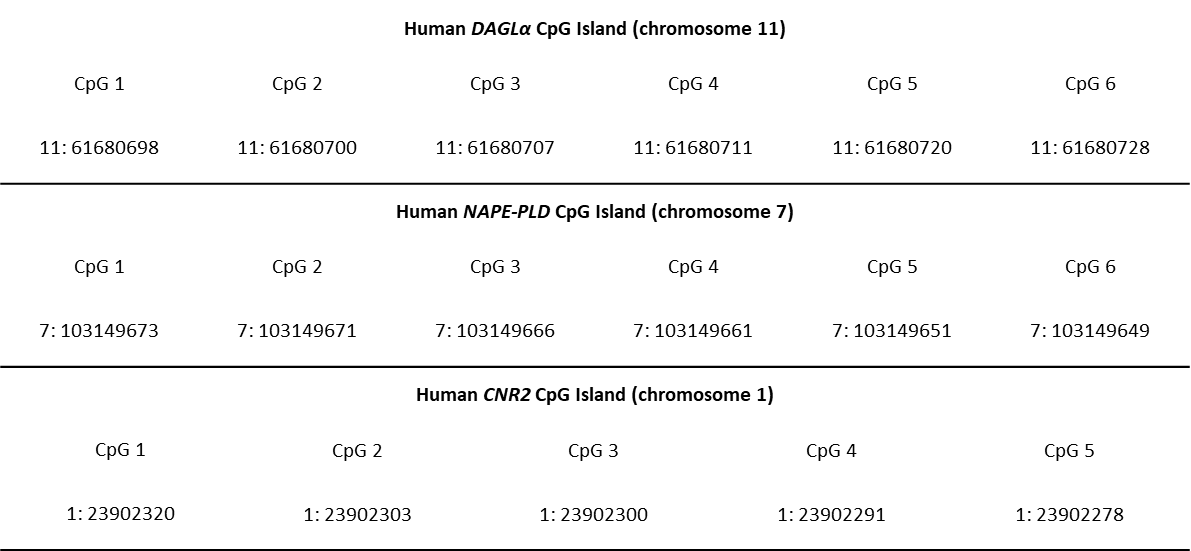


**Supplementary Table 5:**


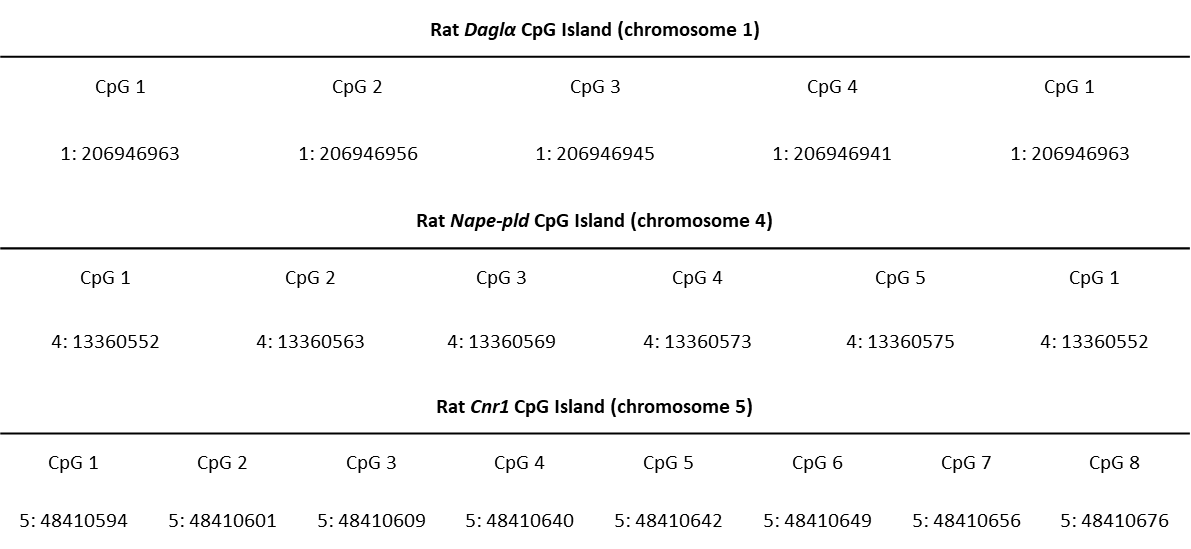


**Supplementary Table 6:**

| **Gene** | **CTRL**  (Mean ± SEM) | **OCD**  (Mean ± SEM) | **p value** |
| --- | --- | --- | --- |
| *CNR1* | 1.35 ± 0.21 | 1.47 ± 0.37 | 0.3682 |
| *CNR2* | **1.09 ± 0.12** | **0.76 ± 0.07** | **0.0263*** |
| *FAAH* | 1.27 ± 0.24 | 0.91 ± 0.15 | 0.9102 |
| *MAGL* | 1.07 ± 0.09 | 1.15 ± 0.12 | 0.7979 |
| *DAGLα* | **1.07 ± 0.08** | **0.74 ± 0.06** | **0.0056**** |
| *NAPE-PLD* | **1.37 ± 0.20** | **0.72 ± 0.15** | **0.0253*** |

**Supplementary Table 7:**

| ***NAPE-PLD***  CpG sites | **CTRL**  (Mean ± SEM) | **OCD**  (Mean ± SEM) | **p value** |
| --- | --- | --- | --- |
| 1 | 5.22 ± 0.19 | 6.41 ± 0.44 | 0.074 |
| 2 | 2.84 ± 0.09 | 3.50 ± 0.28 | 0.135 |
| 3 | 6.31 ± 0.22 | 8.06 ± 0.48 | 0.001 |
| 4 | 3.91 ± 0.13 | 4.65 ± 0.25 | 0.048 |
| 5 | 5.30 ± 0.13 | 6.28 ± 0.34 | 0.088 |
| 6 | 1.08 ± 0.04 | 1.26 ± 0.06 | 0.158 |
| Average | 4.11 ± 0.12 | 5.02 ± 0.30 | 0.020 |

**Supplementary Table 8:**

| ***DAGLα***  CpG sites | **CTRL**  (Mean ± SEM) | **OCD**  (Mean ± SEM) | **p value** |
| --- | --- | --- | --- |
| 1 | 6.96 ± 0.29 | 6.37 ± 0.19 | 0.267 |
| 2 | 6.42 ± 0.35 | 6.34 ± 0.22 | >0.999 |
| 3 | 7.88 ± 1.04 | 6.16 ± 0.22 | 0.530 |
| 4 | 7.64 ± 0.45 | 8.25 ± 0.29 | 0.944 |
| 5 | 7.80 ± 0.27 | 7.65 ± 0.22 | 0.999 |
| 6 | 33.66 ± 0.49 | 32.40 ± 0.56 | 0.554 |
| Average | 11.73 ± 0.25 | 11.24 ± 0.24 | 0.762 |

**Supplementary Table 9:**

| ***CNR2***  CpG sites | **CTRL**  (Mean ± SEM) | **OCD**  (Mean ± SEM) | **p value** |
| --- | --- | --- | --- |
| 1 | 67.29 ± 6.14 | 61.03 ± 7.46 | 0.995 |
| 2 | 76.40 ± 5.67 | 61.58 ± 8.01 | 0.641 |
| 3 | 63.91 ± 6.71 | 64.66 ± 6.85 | 0.999 |
| 4 | 82.44 ± 5.73 | 79.35 ± 5.33 | 0.957 |
| 5 | 55.09 ± 4.52 | 45.96 ± 5.27 | 0.815 |
| Average | 69.03 ± 4.78 | 62.52 ± 5.63 | 0.883 |

**Supplementary Table 10:**

| **Gene** | **CTRL**  (Mean ± SEM) | **MAT-HET**  (Mean ± SEM) | **p value** |
| --- | --- | --- | --- |
| **Prefrontal cortex** | | | |
| *Cnr1* | 1.142 ± 0.18 | 1.011 ± 0.09 | 0.6842 |
| *Cnr2* | 1.214 ± 0.25 | 1.067 ± 0.22 | 0.7197 |
| *Faah* | 1.107 ± 0.14 | 1.196 ± 0.34 | 0.7197 |
| *Magl* | 1.088 ± 0.16 | 1.195 ± 0.31 | 0.4813 |
| *Daglα* | **1.081 ± 0.14** | **0.589 ± 0.12** | **0.0161*** |
| *Nape-pld* | 1.025 ± 0.07 | 1.139 ± 0.26 | 0.9682 |
| **Amygdala** | | | |
| *Cnr1* | **1.148 ± 0.19** | **0.472 ± 0.08** | **0.0041**** |
| *Cnr2* | 1.190 ± 0.25 | 1.332 ± 0.28 | 0.8421 |
| *Faah* | 1.196 ± 0.25 | 0.872 ± 0.14 | 0.6607 |
| *Magl* | 1.192 ± 0.27 | 1.361 ± 0.27 | 0.4967 |
| *Daglα* | 1.070 ± 0.17 | 0.360 ± 0.20 | 0.0789 |
| *Nape-pld* | **1.138 ± 0.20** | **0.616 ± 0.06** | **0.0277*** |

**Supplementary Table 11:**


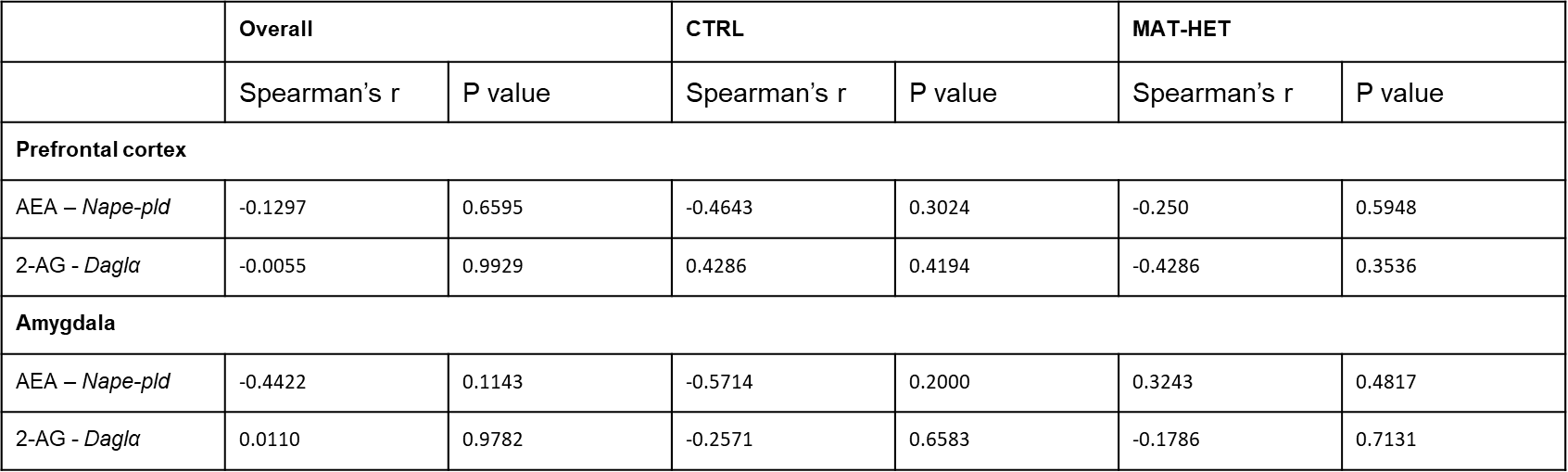


**Supplementary Table 12:**

|  | **CTRL**  (Mean ± SEM) | **MAT-HET**  (Mean ± SEM) | **p value** |
| --- | --- | --- | --- |
| **Prefrontal cortex** | | | |
| *Oxtr* | 1.309 ± 0.53 | 2.09 ± 0.67 | 0.3290 |
| *Bdnf* | 1.048 ± 0.14 | 1.329 ± 0.18 | 0.3290 |
| **Amygdala** | | | |
| *Oxtr* | 1.148 ± 0.19 | 0.472 ± 0.08 | 0.9705 |
| *Bdnf* | 1.323 ± 0.35 | 1.593 ± 0.31 | 0.4470 |
