# Supplementary figures and images for: Selective alterations of endocannabinoid system genes expression in obsessive compulsive disorder
Source: Transl Psychiatry. 2024 Feb 26;14:118. doi: 10.1038/s41398-024-02829-8 (PMC10897168; doi:10.1038/s41398-024-02829-8)

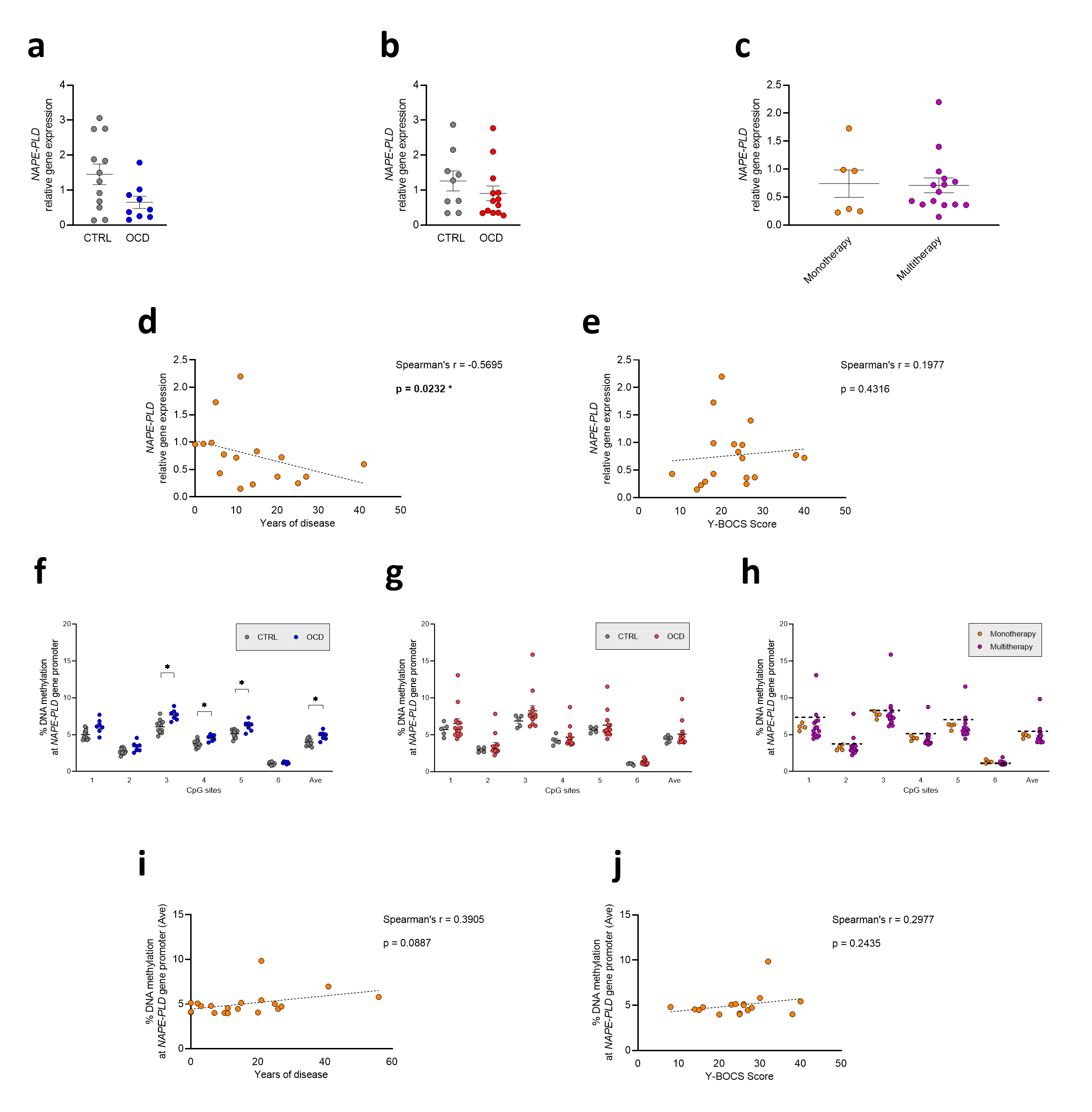

Supplement: Supplementary file 3 — Supplementary Figure 1 [file 41398_2024_2829_MOESM3_ESM.tif]

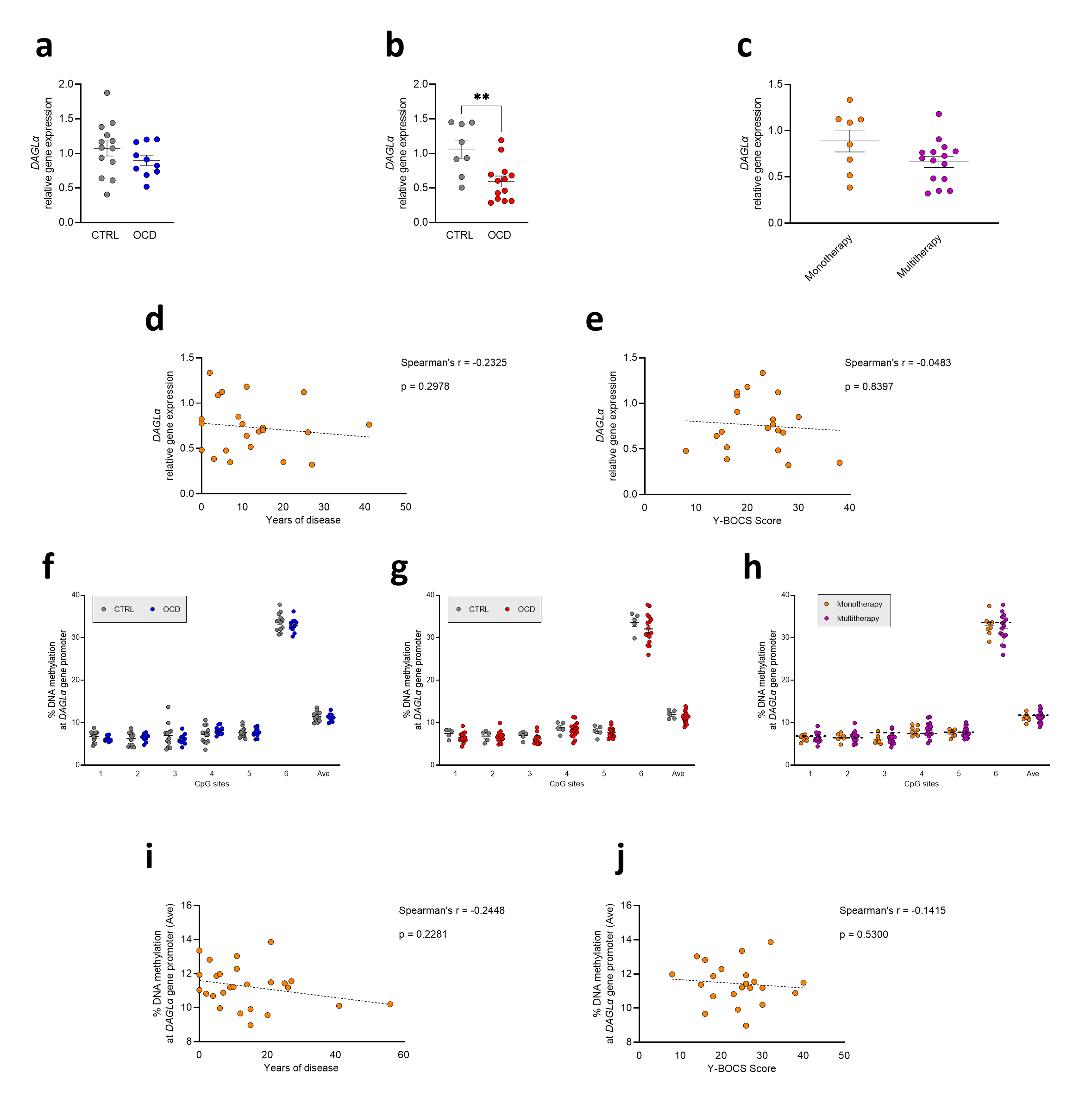

Supplement: Supplementary file 4 — Supplementary Figure 2 [file 41398_2024_2829_MOESM4_ESM.tif]

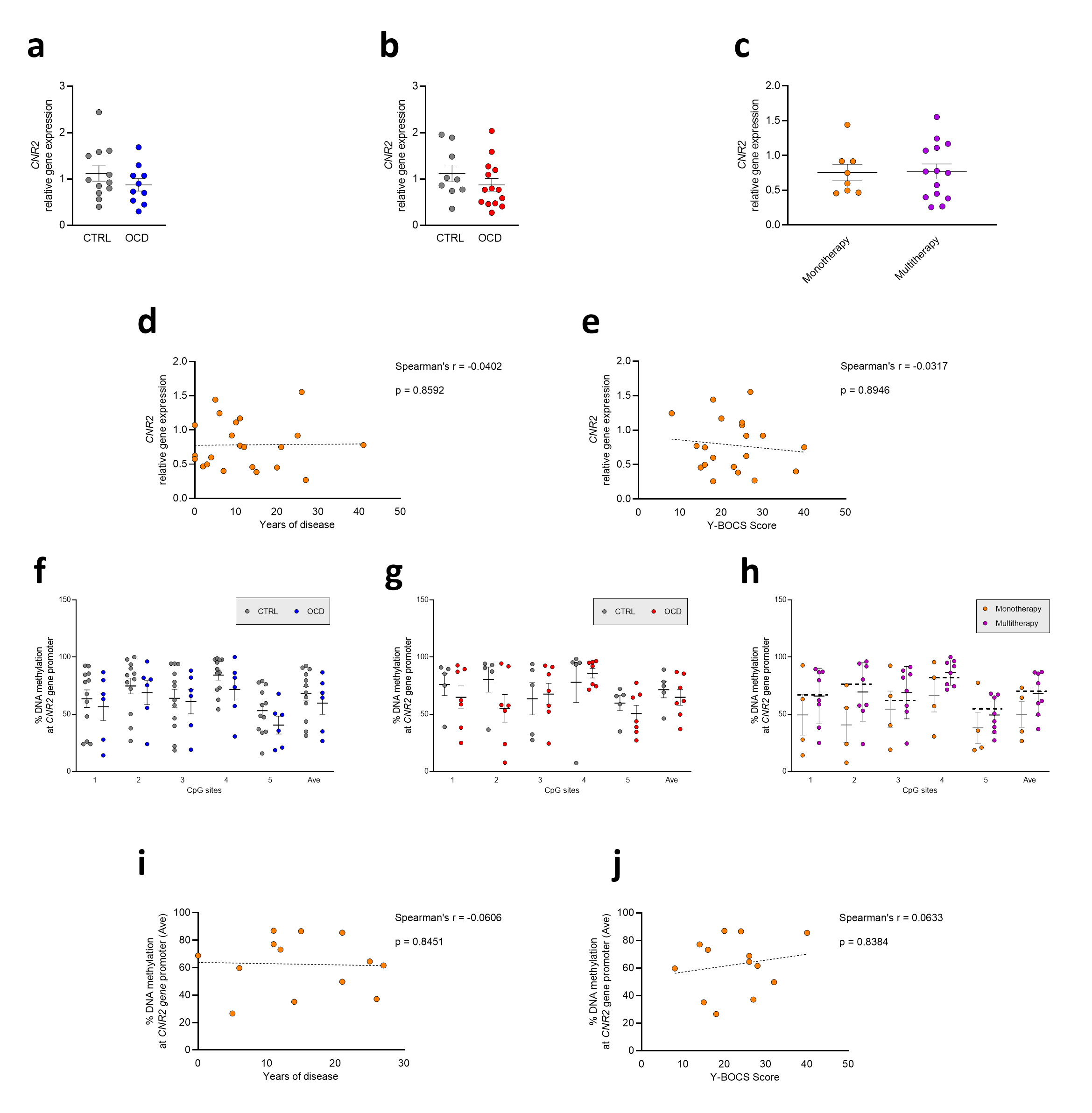

Supplement: Supplementary file 5 — Supplementary Figure 3 [file 41398_2024_2829_MOESM5_ESM.tif]

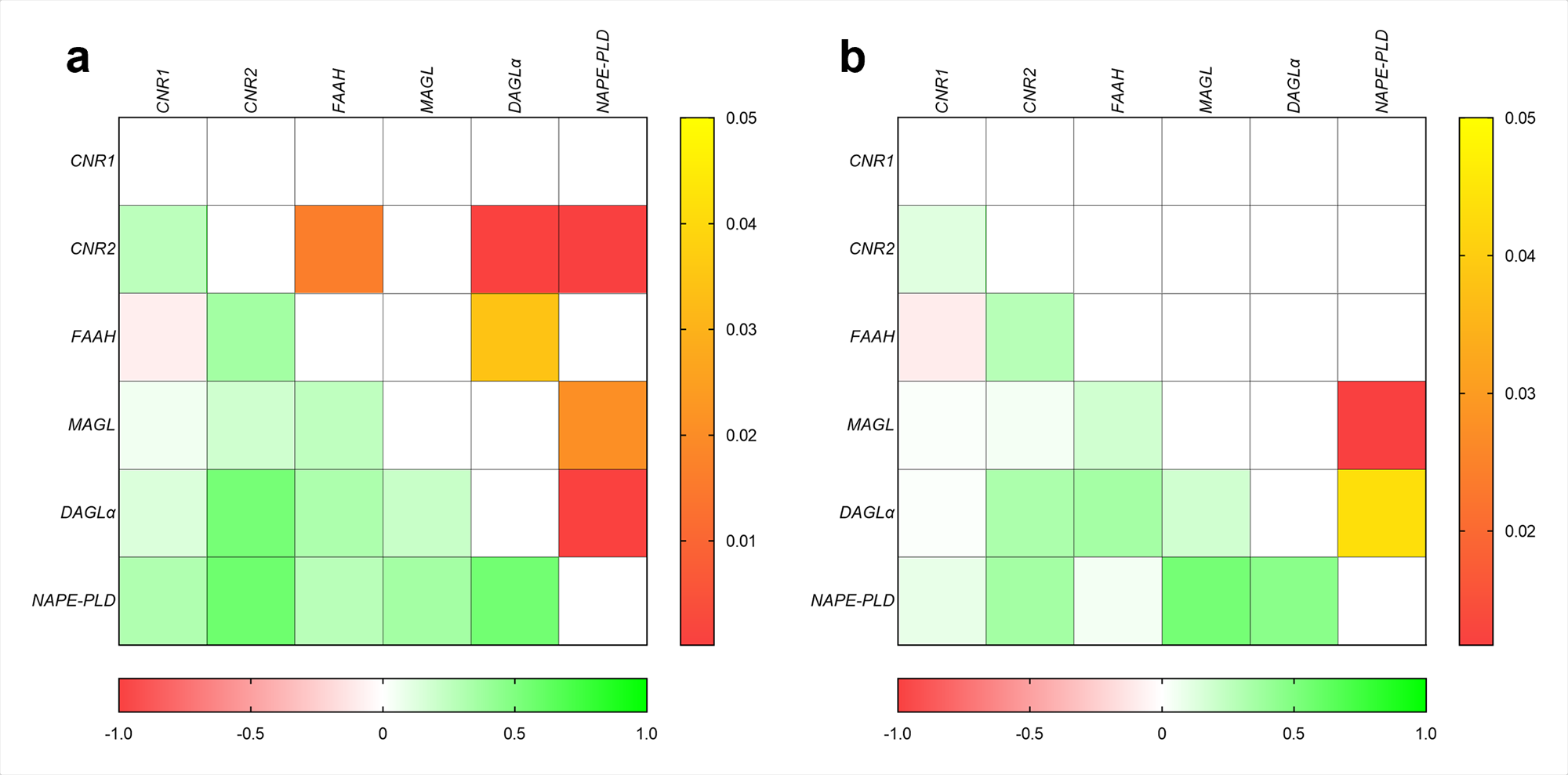

Supplement: Supplementary file 6 — Supplementary Figure 4 [file 41398_2024_2829_MOESM6_ESM.tif]

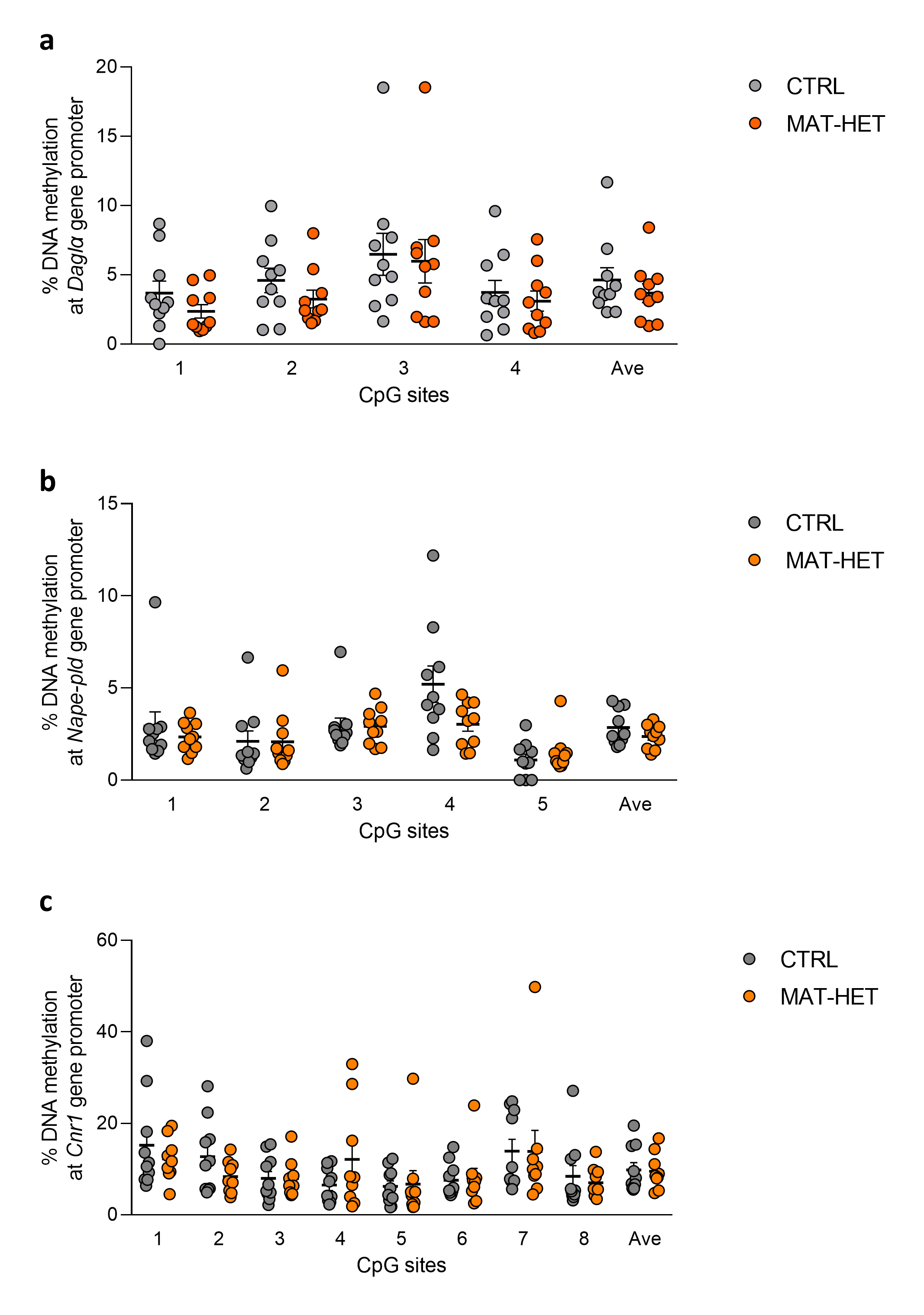

Supplement: Supplementary file 7 — Supplementary Figure 5 [file 41398_2024_2829_MOESM7_ESM.tif]

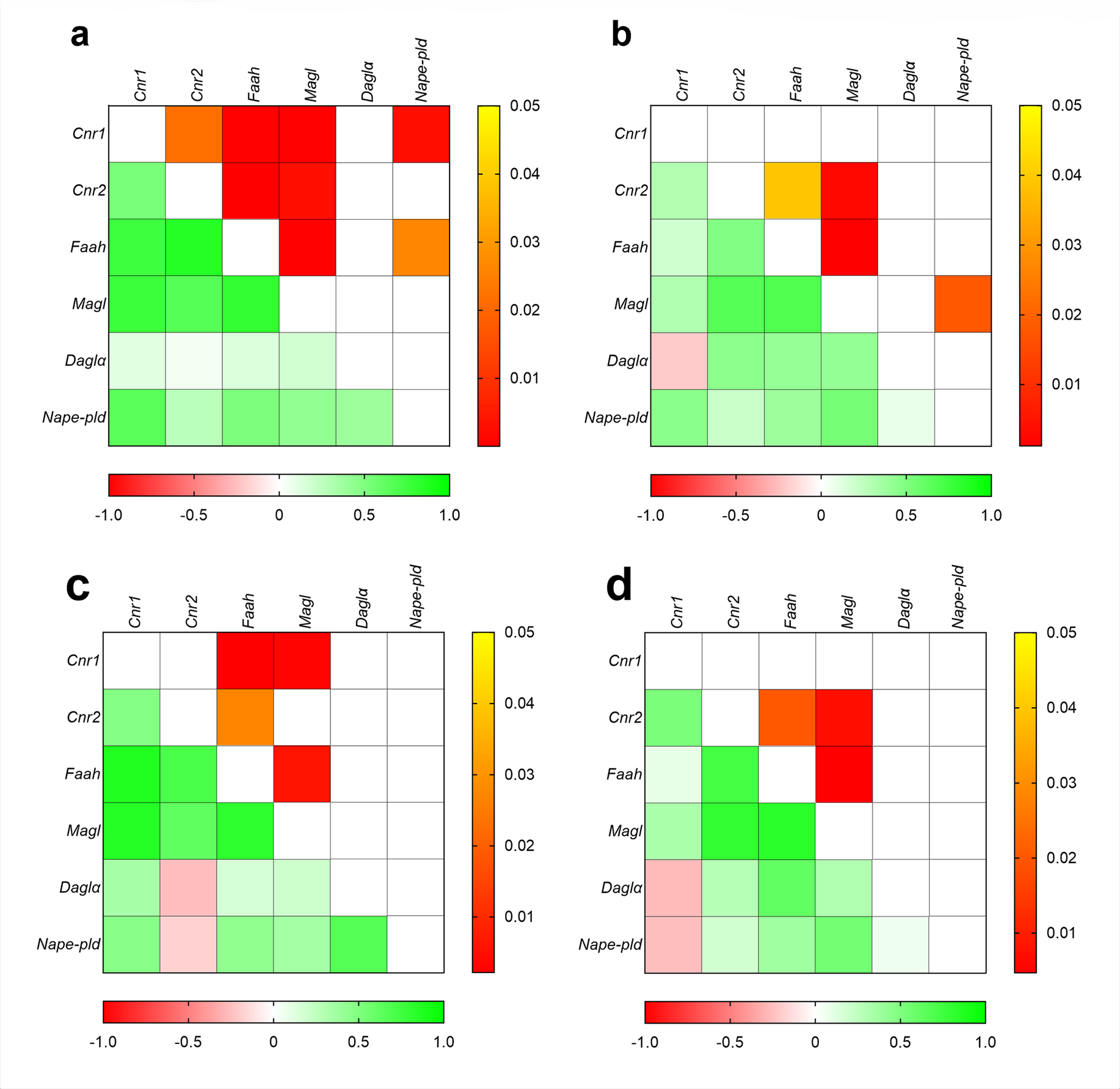

Supplement: Supplementary file 8 — Supplementary Figure 6 [file 41398_2024_2829_MOESM8_ESM.tif]

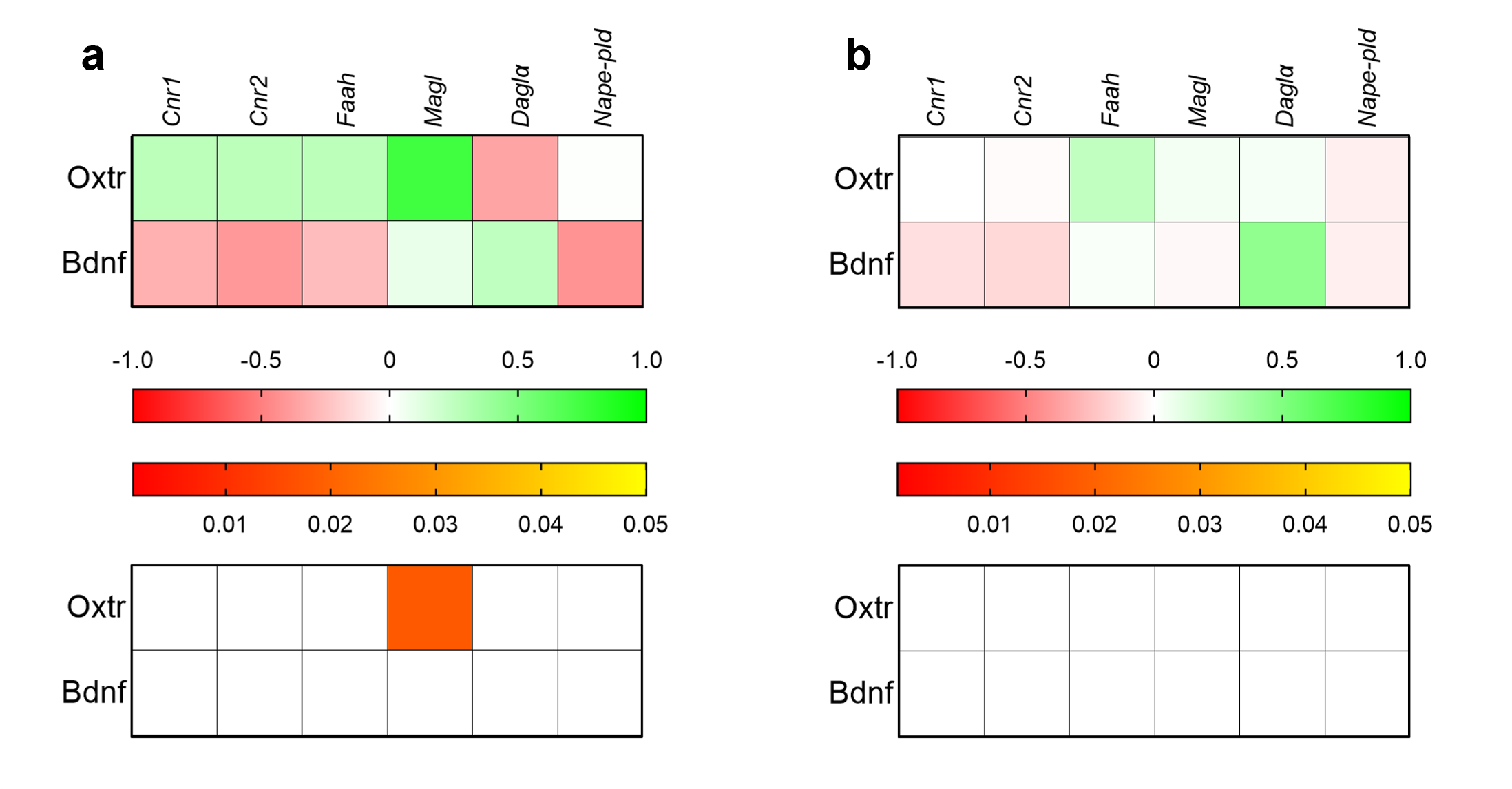

Supplement: Supplementary file 9 — Supplementary Figure 7 [file 41398_2024_2829_MOESM9_ESM.tif]
